# Analysis of gender differences with traditional posterior stabilized versus kinematic designs in total knee arthroplasty

**DOI:** 10.1007/s00402-023-05008-4

**Published:** 2023-08-08

**Authors:** Julian Koettnitz, Jara Tigges, Filippo Migliorini, Christian D. Peterlein, Christian Götze

**Affiliations:** 1 Department of General Orthopaedics, Auguste-Viktoria-Clinic Bad Oeynhausen, University Hospital of Ruhr-University-Bochum, Am Kokturkanal, 32545 Bad Oeynhausen, Germany; 2 Department of Orthopaedics and Trauma Surgery, University Clinic Aachen, RWTH Aachen University Clinic, 52064 Aachen, Germany

**Keywords:** Angle, Complications, Gender, Genesis II, Journey II, Range of motion

## Abstract

**Introduction:**

Total knee arthroplasty (TKA) is a good treatment for end-stage knee osteoarthritis (KOA). Approximately 60% of the patients are females, and 40% are males. This study analyzed pre- and postoperative angle differences in the range of motion (ROM), and the occurrence of complications with traditional posterior stabilization versus kinematic TKA in relation to gender.

**Methods:**

Data from 434 patients with primary cemented total knee arthroplasty from 2018 to 2021 were collected. Alpha and beta angles were determined pre- and postsurgery. The ROM was collected pre- and postoperatively and during follow-up. Additionally, perioperative complications, revision rate, and blood transfusion management were investigated.

**Results:**

The pre- and postoperative alpha-angle between men and women was significantly different, as was the level of alpha-angle correction between men and women (p = 0.001; p = 0.003). Same-gender differences in pre- to postoperative alpha-angles between traditional and kinematic TKA were shown (women (w): p = 0.001; men (m); p = 0.042). High postoperative alpha angles led to less ROM in traditional TKA for women (p = 0.008). No significant gender differences in ROM, perioperative complications, or revision surgery and transfusion rates were found.

**Conclusion:**

Despite high gender differences in pre- and postoperative angles, only female patients with traditional arthroplasty and high postoperative alpha angles showed less ROM in the follow-up. This leads to the assumption that gender-related pre- and postoperative angle differences, and the degree of angle correction, do not influence the ROM or perioperative occurrence of complications. Both designs present safe procedures for both genders with a wide spectrum of axis deformities.

## Introduction

Females are reported to have a higher prevalence of knee osteoarthritis (KOA), leading to a higher number of total knee arthroplasties (TKAs) in women compared to men. The German Joint Replacement Registry Annual Report of 2021 (GJRR) reported that more than 100,000 total knee arthroplasties were performed. Approximately 60% of the patients were female, and 40% were male [1, 2]. TKA is a good treatment for end-stage KOA. Nevertheless, complications repeatedly occur during the perioperative and postoperative course. Many predictors, such as surgeon experience, age, BMI and increased comorbidities, were mentioned [3, 4]. Other intracapsular factors include postoperative varus or valgus imbalances, too tight or loose gap balance in flexion and extension or a high tibial slope. Additionally, Nakano et al. investigated the postoperative knee flexion angle after TKA in relation to the mobility of the hip joint [5–7]. Kinematic Journey II and traditional Genesis II are two commonly used knee implants that have shown promising results in terms of improving patient outcomes and reducing complications following TKA [8]. However, there are limited data on the perioperative sex differences after these two implants. To our knowledge, the consideration of pre- and postoperative alpha and beta angles in relation to ROM, and the rate of perioperative complications and transfusion management in relation to gender differences, have not yet been performed for these two implants.

The aim of this retrospective study was to analyze the perioperative gender differences in patients who underwent TKA using Journey II and Genesis II knee arthroplasty while relating pre- and postoperative alpha and beta angles, ROM, perioperative complications and transfusion management. The hypothesis was that the postoperative angle change would produce gender differences in ROM. Additionally, complications, together with blood transfusions, would be more frequent in one of the genders.

## Methods

### Study design

The present study was performed according to the Strengthening the Reporting of Observational Studies in Epidemiology (STROBE) guidelines [9]. This study was conducted at the Department of Orthopedic Surgery of a University Hospital in Germany. The study was conducted in accordance with the Declaration of Helsinki and approved by the local Ethics Committee.

### Study protocol

**Fig. 1:**
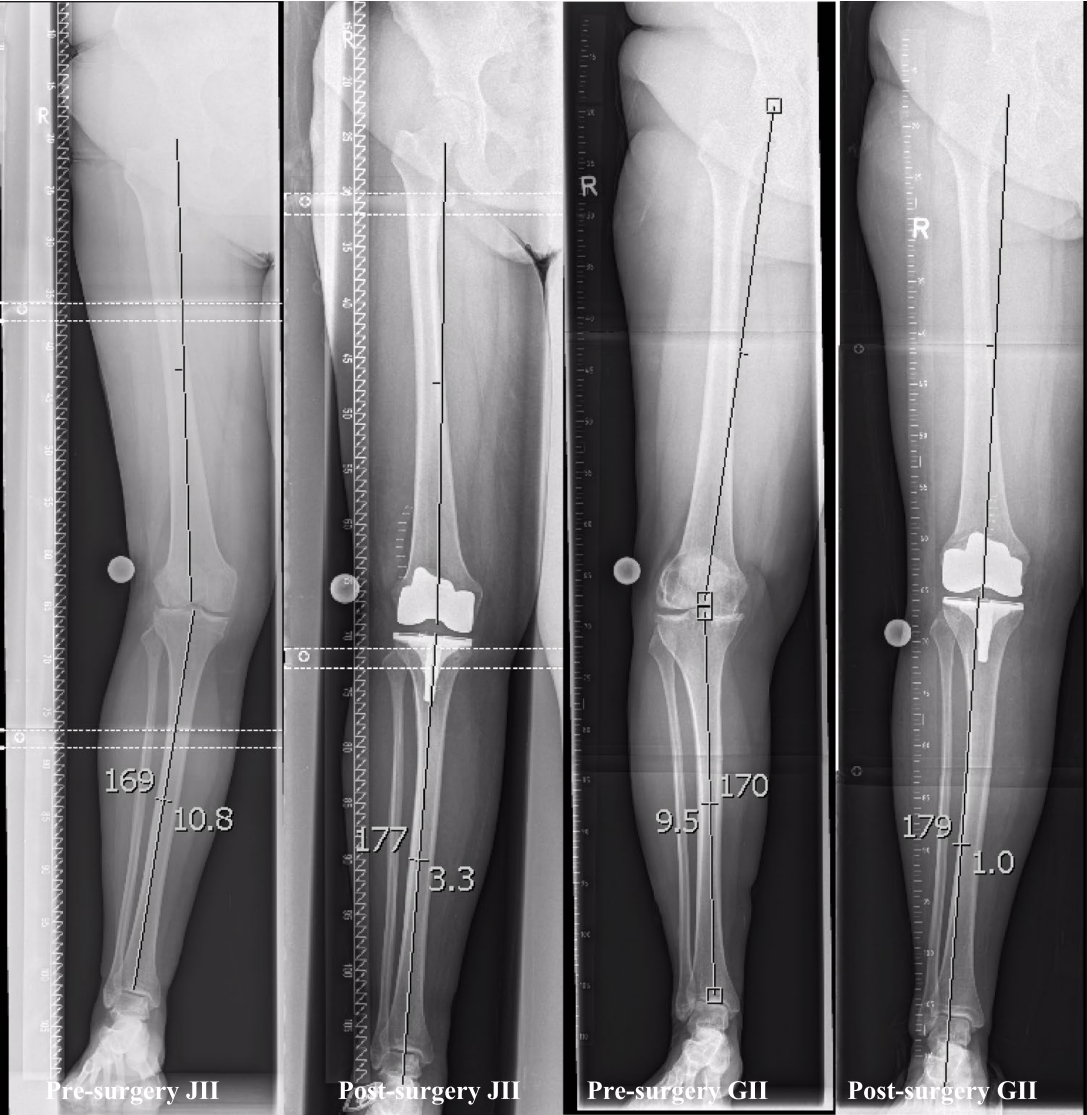
Figures of angle measuring pre- and postoperative with JII and GII arthroplasty; The fixed points were the hip joint center of rotation, the distal femoral center, the proximal tibial center, and the distal tibial center. Valgus angles deviate lateral at the tibial site, varus angles to the medial site;.Measuring depended strongly on the quality of the radiograph;

Data from patients who underwent primary TKA during 2018–2021 were retrieved. Data were retrieved using Nexus Kiss (Nexus Marabu GmbH, Berlin, GE) and collected in Microsoft Excel (Microsoft Corporation, Redmond, US). The following data were collected at admission: age, sex, side, body mass index (BMI), VAS (visual analog scale), alpha and beta angles before surgery, length of hospital stay, and length of intensive care unit stay. The following data were collected during hospitalization: hemoglobin drop requiring transfusion, incidence of systemic and surgical complications, frequency of blood unit transfusions, and alpha and beta angles after surgery. The pre- and postoperative alpha and beta angles were measured using IMPAX Version 6.6.1.3026 (Agfa HealthCare, Mortsel, BG) (Fig. 1). The examiner consecutively reviewed the pre- and postoperative whole-leg radiographs for visibility of the hip, knee, and ankle joints. The alpha and beta angles were then plotted by the examiner. The range of motion was measured through a goniometer from the ward surgeon. Systemic complications included postoperative delirium, urogenital disorders, syncope, nausea and womiting, deep vein thrombosis, resuscitation, electrolyte imbalances, liver malfunction, cardiac complications, patient falls and neurological deficits. Surgical-related complications included acute periprosthetic infection, persistent deficit of ROM, loosening of the arthroplasty, persistent inflammatory signs, postoperative malposition, and wound healing disorder. If patient data were not accessible, the patient was excluded from the present investigation. **(**Fig. 1).

### Eligibility criteria

All patients undergoing primary cemented TKA with Journey II (JII) and Genesis II (JII) arthroplasty were retrieved, and their eligibility was assessed. The inclusion criteria were (1) patients with primary and secondary osteoarthritis, (2) patients aged between 42 and 79 years, and (3) accessible patient data. The exclusion criteria were (1) revision surgery, (2) any blood abnormalities, (3) pregnancy, and (4) peripheral arteriovenous or neurologic ailment.

### Perioperative management

Arthroplasties were performed with Smith & Nephew Genesis II CR/PS and Journey II BCS/CR (S&N GmbH, London, UK). For TKA, a standard medial parapatellar approach was used. Mobilization was forced immediately after surgery. To support postoperative knee mobilization, a motion splint was used every day for 30 min. Stationary or ambulant rehabilitation was organized prior to hospital release.

### Blood unit supply

The indication for blood unit transfusion was according to the restrictive Cochrane guidelines: Hb levels over 8.0 g/dl with no transfusion; Hb levels between 7 and 9 with concomitant clinical symptoms such as dizziness, nausea, malaise or loss of appetite; and Hb levels under 8 g/dl with indicated transfusion [10].

### Statistical analyses

All statistical analyses were performed using the software IBM SPSS version 28 (IBM, Armonk, US). Metric scaled data were analyzed by mean, standard deviation, and variance. Nominal, dichotomous data were analyzed by Fischer’s exact test. Age, BMI and length of hospitalization were listed metrically. Anemia requiring transfusion and frequency of transfusion were listed nominally. Sex, systemic, and surgical complications were listed nominally. For the analysis of metric and nominally scaled variables, the T test for independent samples (including Welch and Levene tests) and variance analyses were used. Cohen`s d (small.20; medium.50; large.80) and 95% interval were used as effect sizes. The effect size used was phi (small.10; medium.30; large 0.50). The significance level was set two-sided with α = 0.05.

## Results

### Recruitment process

Data from 521 patients with primary cemented total knee arthroplasty (Genesis II and Journey II) from 2018 to 2021 were retrieved. Eighty-seven patients were excluded because they were older than 79 years. Finally, data from 434 patients were collected: 267 Genesis II and 163 Journey II.

### Patient demographics

Data from 434 patients were collected. A total of 37.8% (164 of 434) were men, and 62.2% (270 of 434) were women. In the Journey II group, there were 18.2% (79 of 434) men and 20.3% (88 of 434). In the Genesis II group, there were 19.6% (85 of 434) men and 41.9% (182 of 434) women. The mean BMI for men was 31.51 ± 6.24. The mean BMI for women was 31.92 ± 7.0.

The mean age of the men was 67.8 ± 7.8 years. The mean age of the women was 68.0 ± 8.1 years. For men, the time of operation was 103 ± 43 min, and for women, it was 96 ± 29 min. The length of hospitalization for men was 9.74 ± 301 days, and for women, it was 10.01 ± 2.86 days.

### Angle and range of motion

Angle: For preoperative alpha, n = 155 men, n = 255 women and for postoperative alpha, n = 133, n = 196 were investigated. Preoperative beta n = 154 men and n = 249 women and postoperative beta n = 131 men and n = 191 women were investigated. The pre- and postoperative alpha-angle between men and women was significantly different (Cohen’s d = 0.486; p = 0.001; Cohen’s d = 0.411; p = 0.001). There was a significant difference in the level of alpha-angle correction between men and women (Cohen’s d: 0.325; p = 0.003). The mean pre- and postoperative beta-angle was significantly different (Cohen’s d: 0.228; p = 0.021; Cohen’s d: 0.233; p = 0.032) between males and females (Table 1). The preoperative alpha and beta angles of women with JII and GII arthroplasty were significantly different (Cohen’s d = 0.396, p = 0.002; Cohen’s d = 0.319, p = 0.030). The postoperative alpha and beta angles of women with JII and GII were not different (Cohen’s d = 0.134, p = 0.353; Cohen’s d = 0.037, p = 0.822). The difference in pre- and postoperative alpha angle between women with JII and GII arthroplasty was significant (Cohen’s d = 0.492, p = 0.001). For men with JII and GII, there was no significant difference in the preoperative alpha and beta angle and postoperative beta angle (Cohen’s d = 0.138, p = 0.390; Cohen’s d = 0.047, p = 0.771; Cohen’s d = 0.098, p = 0.572), but there was a significant difference in the postoperative alpha angle and the change in pre- to postoperative alpha angle (Cohen’s d = 0.477, p = 0.007; Cohen’s d = 0.358, p = 0.042) Table 2.

**Table 1:** Gender differences in pre- and postoperative angle

Gender/angle	Preoperative	Postoperative
Men (alpha)*	6.03° ± 7.03°	3.32° ± 3.22°
Women (alpha)*	1.99° ± 8.97°	1.93° ± 3.47°
Men (beta)*	5.01° ± 2.58°	5.81° ± 1.63°
Women (beta)*	4.35° ± 3.08°	5.35° ± 2.13°

Mean ± SD; *significantly different pre- postoperative angle between men and women

**Table 2:** Same-gender analysis of pre- and postoperative alpha and beta angles; mean ± SD

	Men				Women			
	Alpha		Beta		Alpha		Beta	
	JII	GII	JII	GII	JII	GII	JII	GII
Pre-surgery	5.56° (± 7.55°)	6.53° (± 6.43°)	4.96° (± 2.94°)	5.08° (± 2.13)	− 0.119° (± 8.79)	3.38° (± 8.85°)	3.79° (± 3.91°)	4.73° (± 2.35°)
Post-surgery	4.00° (± 3.17°)	2.50° (± 3.12°)	5.88° (± 1.72)	5.72° (± 1.52)	2.21° (± 3.42)	1.73° (± 3.5°)	5.31° (± 2.87°)	5.39° (± 1.39°)

Mobility: For ROM preoperative n = 159 men, n = 264 women, postoperative n = 157 men, n = 245 women and at reappointment n = 75 men and n = 133 women were measured. The preoperative ROM differed significantly between men and women (Cohen’s d = 0.288; p = 0.004). There was no significant difference in the postoperative and follow-up ROM (Cohen’s d = 0.125, 0.066; p = 0.221, 0.651). There was no significant difference in the postoperative and follow-up ROM of men and women with GII and JII arthroplasty (JII postoperative/follow-up: Cohen’s d = 0.029, p = 0.859; Cohen’s d = 0.008, p = 0.971; GII postoperative/follow-up: Cohen’s d = 0.141, p = 0.304; Cohen’s d = 0.117, p = 0.54). There was no significant difference in same-gender analyses of pre, postoperative and reappointment ROM between GII and JII arthroplasty (men: Cohen’s d = 0.129, 0.044, 0.298; p = 0.413, 0.783, 0.197; women: Cohen’s d = 0.008, 0.026, 0.174; p = 0.953, 0.849, 0.333). At reappointment, a significant correlation between high postoperative alpha angles and less ROM with GII arthroplasty for women was found (Pearson-correlation = -0.395, p = 0.008). JII for females and GII and JII for males did not show any significant correlation with ROM at reappointment.

### Complications and revisions

For 9.75% (16 of 164) of men and 8.51% (23 of 270) of women, systemic complications occurred. Surgical complications occurred in 3.65% (6 of 164) of the male cases and in 2.70% (13 of 270) of the female cases. One male patient (0.6%) showed surgical and systemic complications at once. A total of 3.65% (6 of 164) of the male patients received two revisions. A total of 2.22% (6 of 270) of the female patients received a revision. No female patient received two revisions (see Table 3).

**Table 3:** *ROM and gender; ROM was assessed through the full possible range of motion in degrees; mean* ± *SD*

ROM/Gender	Women	Men
Preoperative	98.41° ± 16.16°	102.98° ± 15.51°
Postoperative	88.67° ± 5.46°	89.33° ± 4.87°
Reappointment	104.77° ± 19.83°	106.01° ± 16.87°

### Anemia and blood unit transfusion

A total of 5.48% (9 of 164) of the males and 11.85% (32 of 270) of the females suffered from anemia requiring blood transfusion. A total of 4.2% (7 of 164) of the male patients and 6.2% (17 of 270) of the female patients received blood transfusions (Table 3, 4).

**Table 4:** Analysis of sex differences in the occurrence of systemic and surgical complications, revision surgeries, anemia and transfusion management. Revision surgery included head-inlay changes, wound revisions, inlay + component changes, inlay changes and wound revisions

	phi	p
Systemic and surgical complications	0.206	0.611
Revision surgery	0.075	0.284
Anemia requiring Transfusion	0.105	0.028
Blood transfusion	0.109	0.109

## Discussion

This study investigated gender differences in pre- and postoperative alpha- and beta angles, the relation to ROM, and the occurrence of complications and blood transfusion with traditional and kinematic TKA. The pre- and postoperative alpha and beta angles were significantly different between males and females. In addition, the correction of the alpha angle was significantly different between men and women. No differences between GII and JII arthroplasty for men were found in alpha and beta angles pre- and postoperatively. Women revealed a significant correlation between a high postoperative alpha-angle and less ROM at reappointment. Pre- and postoperative ROM did not show any significant differences between males and females. No gender differences were found for complications and transfused blood. Females were more likely to have anemia requiring blood transfusion.

In terms of complications, these findings are different from other studies, where gender differences were shown for the risk of wound infections or reoperation, length of stay, fractures and survivorship [11–14]. Alternatively, Crawford et al. 2023 did not reveal any sex differences in the long-term survival rate of unicompartmental knee arthroplasty. For the occurrence of blood transfusion, similar results with no gender differences were shown in the past [15, 16]. Interestingly, in this study, females were more likely to have anemia requiring blood transfusion, although this did not lead to increased transfusion rates. As the rates of blood transfusion, complications, and revision surgery were similar between sexes, anemia < 8.0 g/dl should not be used as a predictor for complications. Overall, the study showed that primary TKA is a safe procedure for males and females. Similar revision rates of approximately 3% were found in the literature [17, 18]. Analyzing gender differences in pre- and postoperative alpha and beta angles revealed gender differences as well as same-gender differences. In general, men presented higher alpha angles presurgery and a higher difference in postoperative alpha angles. The beta angles in both sexes were higher postoperatively than preoperatively and significantly different between men and women. A deeper view on gender dissimilarities between the implant designs and men or women revealed differences in pre- to postoperative alpha angles for men and women, whereas the postoperative results were only different for men. Significant connections to the postoperative ROM at reappointment could be found for women with GII arthroplasty. Higher postoperative angles led to worse ROM in the follow-up. In contrast, McNamara et al. 2022 did not show a different outcome between GII and JII arthroplasty after 6 months. Additionally, a better outcome in the walking range of motion was found [19]. Shichman et al. revealed no differences between traditional and kinematic TKA in a follow-up of 2 years. The postoperative ROM was almost identical with 113° flexion, and the revision rate was not significantly different [20]. In comparison to these investigations, no gender differences were found, and the age of patients was not limited. This raises the question of whether gender differences are dependent on the age of patients. In summary, despite great pre- and postoperative differences in alpha and beta angles for men and women, the effect on postoperative ROM is limited. A view to other studies, such as Scott et al. 2023, shows that there was also no gender difference in stability and ROM. Differences were found only for the type of implant [21]. Other investigations described a difference in postoperative satisfaction between males and females, as well as negative correlations between female gender and postoperative range of motion [22, 23].

This study has several limitations. The retrospective design is limited to a direct follow-up, depending on rescheduled appointments. In-hospital data are often lacking due to limited archive files. The gender distribution between men and women was 60%. Surgery was performed by more than 10 different surgeons with different skill levels, which could influence the data. Measurements of the pre- and postoperative angle depended on a single examiner and a single radiographic image viewing software. The degree of ROM was assessed by several surgeons of the hospital.

## Conclusion

In conclusion, gender differences and corrections were found for the pre- and postoperative degrees of alpha and beta angles, as well as the same gender differences in the corrections of alpha and beta angles between GII and JII arthroplasty. Only female patients with GII arthroplasty and high postoperative alpha angles showed less ROM in the follow-up. Transfusion requiring anemia occurred more frequently among women. However, no significant differences were found for surgical revisions, rate of complications, or blood supply. This leads to the assumption that gender-related pre- and postoperative angle differences, as well as the degree of angle correction, do not influence the ROM or perioperative occurrence of complications. GII and JII arthroplasty appear as safe procedures for both genders with a wide spectrum of axis deformities.

## Data Availability

All data are available at the corresponding author upon request.
